# Recent advances of myotubularin-related (MTMR) protein family in cardiovascular diseases

**DOI:** 10.3389/fcvm.2024.1364604

**Published:** 2024-03-11

**Authors:** Jia Wang, Wei Guo, Qiang Wang, Yongjian Yang, Xiongshan Sun

**Affiliations:** ^1^ Department of Cardiology, The General Hospital of Western Theater Command, Chengdu, Sichuan, China; ^2^ College of Medicine, Southwest Jiaotong University, Chengdu, Sichuan, China; ^3^ Clinical Research Center, Stomatological Hospital of Chongqing Medical University, Chongqing, China

**Keywords:** cardiovascular diseases, MTMR, phosphoinositide, PI3K/AKT, autophagy

## Abstract

Belonging to a lipid phosphatase family containing 16 members, myotubularin-related proteins (MTMRs) are widely expressed in a variety of tissues and organs. MTMRs preferentially hydrolyzes phosphatidylinositol 3-monophosphate and phosphatidylinositol (3,5) bis-phosphate to generate phosphatidylinositol and phosphatidylinositol 5-monophosphate, respectively. These phosphoinositides (PIPs) promote membrane degradation during autophagosome-lysosomal fusion and are also involved in various regulatory signal transduction. Based on the ability of modulating the levels of these PIPs, MTMRs exert physiological functions such as vesicle trafficking, cell proliferation, differentiation, necrosis, cytoskeleton, and cell migration. It has recently been found that MTMRs are also involved in the occurrence and development of several cardiovascular diseases, including cardiomyocyte hypertrophy, proliferation of vascular smooth muscle cell, LQT1, aortic aneurysm, etc. This review summarizes the functions of MTMRs and highlights their pathophysiological roles in cardiovascular diseases.

## 1 Introduction

As a second messenger within the cell, phosphoinositides (PIPs) participate in a variety of cellular processes, such as protein transport, signal transduction, remodeling of the protein backbone, and fusion of the cell membrane. PIPs can be hydrolyzed into several kinds of substrates, phosphatase and tensin homolog deleted on chromosome ten (PTEN), myotubularin-related (MTMR) protein family, sac1 domain-containing phosphatase, etc. (1, 2). MTMR protein family consists of 16 members, of which 9 members are active phosphatases, while the rest 7 members are inactive phosphatases due to lacking of the conserved cysteine in the catalytic signature (2–4). The most well-known and distinguished effect of MTMRs is the ability to dephosphorylate phosphatidylinositol 3-monophosphate (PI(3)P) and phosphatidylinositol (3, 5) bis-phosphate (PI(3,5)P2), indicating MTMRs are involved in cellular membrane transport and endocytosis (5). MTMRs are widely expressed in different kind of tissue and organs, including the neural system, heart, liver, testicle and gastrointestinal tract (6–8). MTMRs also exert multiple physiological roles such as modulating cell proliferation, differentiation, necrosis and migration (9). Recent studies demonstrate that several MTMRs are also involved in the development of CVDs (10, 11). Thus, illuminating the role of MTMRs in cardiovascular system is of great importance to search novel targets for preventions of CVDs. This review highlights the function of MTMRs in the cardiovascular system and discusses the associated mechanisms.

## 2 Overview of the canonical physiological mechanisms of MTMRs

### 2.1 Interactions between MTMRs

MTMRs have been found to have several functional domains that mediate the interactions of protein-protein and protein-lipid, such as the PH-glucosyltransferase, Rab-like GTPase activator and myotubularin (GRAM) domain is involved in the interaction with membranes, Rac-induced recruitment domain which is a membrane-targeted motif, SET interacting domain/PDZ binding domain which mediate protein-protein interactions, and Zinc FYVE domain coupled with phosphatidylinositol (2, 12). Furthermore, the coiled-coil (CC) domain is essential for the homodimerization or heterodimerization between MTMRs (13). The nine members of MTMR with catalytic activity can interact with other members without enzymatic activity, and this interaction between MTMRs plays a crucial role in maintaining its normal function (Table 1). Nandurkar et al. demonstrate that MTMR12, also known as 3-phosphatase adaptor (3-PAP), is a catalytically inactive member of MTMR family and can interact with myotubularin (MTM1) and MTMR2. Co-expression of catalytically inactive MTMR12 with MTM1 can reverse the remodeling of membrane phenotype caused by overexpression of MTM1, translocate MTM1 into cytoplasm, and also attenuate the formation of filamentous pseudopodia caused by overexpression of MTM1 (14). The interaction between MTM1 and MTMR12 is essential for the stability of functional protein complexes in skeletal muscle, which offers novel targets for *Mtm1* mutation-induced X-linked myotubular myopathy (XLMTM) (15). MTMR2 is a 73-kDa protein that forms a dimer via its coiled structure, while its interacting partner, MTMR13/SBF2 belongs to the catalytically inactive members. Mutation of either MTMR2 or MTMR13 leads to Charcot-Marie-Tooth type 4B, which is characterized by reduced nerve conductive velocity and folding of myelin within the peripheral nerve (16). MTMR2 binds to MTMR13 and forms a protein complex within Schwann cell, which is critical for the integrity of peripheral nervous system (17). Additionally, Kim et al. demonstrate that MTMR2 interacts with MTMR5 via its CC domain, which enhances the enzymatic activity of MTMR2 and alters its subcellular localization (5).

**Table 1:** Several MTMRs can homodimerize or heterodimerize to form active-active or active-inactive complexes, allowing more precise regulation of phosphoinositide activity.

Name		Homodimerization with	Heterodimerization with
MTM with catalytic activity	MTM1	√	MTMR12
	MTMR1		MTMR12
	MTMR2		MTMR5, MTMR13
	MTMR3	√	MTMR4
	MTMR4	√	
	MTMR6	√	MTMR9
	MTMR7		MTMR9
	MTMR8		MTMR9
	MTMR14/JUMPY		
MTM with no catalytic activity	MTMR5/SBF1		MTMR2
	MTMR9/LIP-STYX	√	MTMR6, MTMR7, MTMR8
	MTMR10		
	MTMR11/CRAa/b		
	MTMR12/3-PAP	√	MTM1, MTMR1, MTMR2
	MTMR13/SBF2	√	MTMR2
	MTMR15/FAN1		

In addition to heterologous interaction between catalytically active and inactive MTMRs, two catalytically active MTMRs can also interact with each other. The first reported interaction between two catalytically active MTMRs was MTMR3-MTMR4 (18). A subfamily of homologous MTMRs include MTMR6, MTMR7, and MTMR8, all of which can form a heterodimer with MTMR9. The MTMR6/MTMR9 complex has higher activity against, while the MTMR8/MTMR9 complex prefers PI(3)P as substrate (19). Some cellular processes have been implicated in the function of MTMR6/7/8/9 myostuin subsets, such as the heterodimer of MTMR6-MTMR9, which is confirmed to ameliorate cellular apoptosis both *in vivo* and *in vitro* (20).

### 2.2 Modulating the PI3K/AKT pathway

PI3K/AKT pathway plays a vital role in cardiovascular events, including atherosclerosis, cardiac hypertrophy, and vascular remodeling (21). AKT can be activated by a variety of external stimuli in cardiovascular system, such as insulin, vascular endothelial growth factor (VEGF), reactive oxygen species (ROS), and several phosphatase inhibitors (22, 23). These stimuli usually transcriptionally or post-translationally regulate AKT activity. AKT exerts its roles in cardiovascular system by affecting its downstream targets. For example, AKT induces endothelial nitric oxide synthase (eNOS) phosphorylation, vasodilation, and angiogenesis via enhancing VEGF secretion. AKT promotes cellular survival via suppressing FOXOs, caspase 9, and Bcl-2. Additionally, AKT also induces cellular growth and proliferation via increasing mammalian target of rapamycin complex 1 (mTORC1) activity (21).

**Figure 1:**
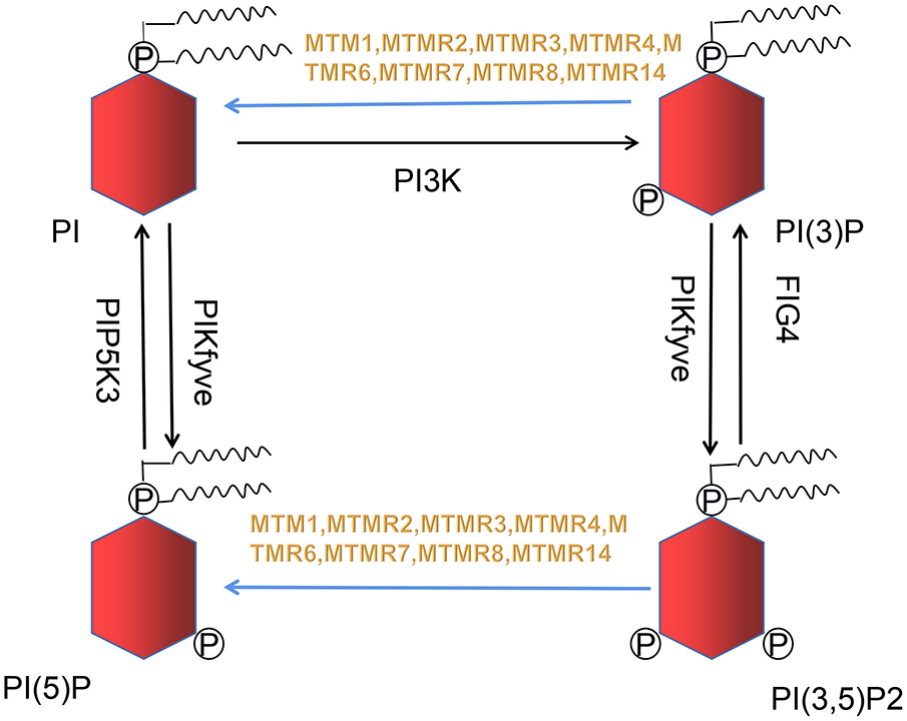
Nine out of sixteen members of the MTMRs family possess catalytic activity, dephosphorylating PI(3)P and PI(3,5)P2 to PI and PI(5)P. This metabolic reaction is indicated in blue arrow.

Razidlo et al. demonstrate that silencing MTM1 significantly inhibits growth factors-induced AKT phosphorylation, which is resulted from abnormal accumulation of PI(3)P, the substrate of MTMR (24). PI(3)P and PI(3,5)P2 generate into phosphatidylinositol (PI) and phosphatidylinositol 5-monophosphate (PI(5)P) when MTM1 is dephosphorylated (Figure 1), while MTM1 knockdown causes a 2-fold rise in total PI(3)P in cell (25). PI(3)P is usually believed to originate from phosphatidylinositol kinase type III. However, increasing evidences suggest that PI3K-C2β can also generates PI(3)P. Overexpression of PI3K-C2β suppresses AKT phosphorylation within mammalian cell, indicating that impaired AKT phosphorylation may be caused by excessive accumulation of PI(3)P (26).

### 2.3 Physiological function description of atypical MTMR

The specific pathological and physiological mechanisms of atypical MTMR are as follows. Among the genes abnormally expressed in ankylosing muscular dystrophy type 1 (DM1), myosin associated 1 gene (MTMR1) is associated with impaired muscle differentiation. Maria et al. found that her2 can regulate MTMR11 and promote malignant tumor proliferation. The human heterozygous 15q13.3 microdeletion contains genes FAN1/MTMR15 and MTMR10, which are associated with neuropathological disorders. In addition, MTMR15, as a highly conserved protein, MTMR15/FAN1, interacts with the monoubiquitinated form of FANCD2 and recruits DNA damage sites through FANCD2, promoting repair.

## 3 Roles of MTMRs in cardiovascular system

There are growing evidences that some MTMRs are differentially expressed in cardiovascular diseases (Table 2). For instance, MTM1 is highly expressed in the membrane of platelets and utilized in the diagnosis of X-linked myotubular myopathy (XMLM). MTMR4 has an effect on the development of LQT1 and aortic aneurysm. As a positive regulator of peroxisome proliferator-activated receptor gamma (PPARγ), MTMR7 has certain diagnostic and therapeutic value for the prevention and treatment of heart failure. MTMR14 regulates cardiomyocyte hypertrophy and proliferation of vascular smooth muscle cell.

**Table 2:** Summary of the pathophysiological roles of MTMRs in cardiovascular diseases and common biological functions.

Myotubularins	Application in the cardiovascular system	Universal physiological functions
MTM1	Distributed at the membrane of platelets and co-localized with α granule	Increase the amount of autophagosome
	Absence leads to cholestatic liver disease and may be associated with increased cardiovascular risk factors in children with cholestatic disease	
MTMR3/MTMR4	Prevents protein aggregation in trophoblasts and abnormal placental dysfunction in preeclampsia	Regulates autophagy
	Alleviate the clinical manifestations of LQT1 patients	
	May be a potential therapeutic target in the process of myocardial fibrosis	
MTMR7	Regulates glucose and fatty acid metabolism	Negative regulation of autophagy
	May be a potential research target for the relationship between vCJD and cardiovascular disease	
MTMR14	Regulates myocardial hypertrophy	Regulates lipid metabolism
	Regulates myocardial contractility	Inhibits basal autophagy
	Inhibits the proliferation of vascular smooth muscle cells	
MTMR2/MTMR5		Inhibits autophagy
MTMR6	May be possible to modulate cardiovascular events by modulating KCa3.1	Actively regulates late autophagy
		Regulates potassium channels
MTMR9	SNP RS2293855 on the MTMR9 gene intron is associated with increased HbA1c level	Regulates lipid metabolism
		Interacts with MTMR8 to reduce autophagy activity

CVDs, cardiovascular diseases; LQT1, long QT syndrome; LQT1, subtype; vCJD, variant Creutzfeldt-Jakob diseases; KCa3.1, Ca^2+^-activated K^+^ channels3.1.

### 3.1 MTM1

Molecular genetics and histopathology are presently used to diagnose XMLM, which can be resulted from *Mtm1* mutation (27). MTM1, a hyperactive 3-phosphatase, is discovered to be abundantly expressed in platelets. MTM1 is mainly distributed at the membrane of platelets and co-localized with α granule. Furthermore, there is no change in aggregation and secretory reaction of platelets after stimulus of thrombin or collagen by using a mouse *Mtm1*-knocking out model, suggesting that other MTMRs instead of MTM1 play a role in platelets (28). In fact, the mRNA levels of several members of MTMRs are increased during the process of human hemopoietic progenitor cells differentiate into megakaryocyte (29). Whether which kind of MTMR functions in aggregation and secretory reaction of platelet needs further exploration.

MTM1 can interact with a protein which is a striated muscle preferentially expressed protein kinase (SPEG) (30). SPEG plays an important role in the excitation-contraction coupling (31), cytoskeleton organization (32), and other cellular processes (33). The *Speg* gene is recessively mutated in central nuclear myopathy (CNM) and dilated cardiomyopathy (30). Embryonic *Speg*-KO mouse shows cardiac enlargement, cardiac fibrosis, decreased cardiac function after birth, and eventually died, which confirms the relationship between SPEG and the occurrence of dilated cardiomyopathy (34). Whether MTM1 plays a role in the pathogenesis of dilated heart disease needs to be further explored. The most recent report was that in 2023, Ka et al. described in a model of zebrafish that loss-of-function mutations in MTM1 lead to severe cholestatic liver disease, while previous studies have reported that there may be increased cardiovascular risk factors in children with cholestatic disease, suggesting that MTM1 may be involved in the occurrence and development of cardiovascular events (35).

### 3.2 MTMR3/MTMR4

Yeast two-hybrid and co-immunoprecipitation experiments have shown that MTMR3 can bind to MTMR4, so MTMR3 and MTMR4 are introduced together (18). that MTMR3 was found to be a direct target of miR-181a, linking the miRNA to autophagy. In turn, the increase of miR-181a reduced MTMR3, inhibited the occurrence of autophagy, prevented protein aggregation in trophoblasts and abnormal placental dysfunction, and provided a potential therapeutic target for the diagnosis of preeclampsia (36), thereby further reducing the risk of cardiovascular disease, diabetes and other metabolic diseases in women and infants who survived preeclampsia (37).

MTMR4 is a 133-kDa intracellular protein and has two single nucleotide variations (SNVs) within its conserved phosphatase region, which attenuates the degradation of channel proteins and protects ion channels (38). Congenital long QT syndrome (LQTS) is the first reported channelopathy and is associated with mutations of genes encoding ion channels or their regulatory proteins, among which LQT1 is the most common one (39). MTMR4 is confirmed to alleviate the clinical manifestations of LQT1 patients due to the existence of SNVs, which also explain why LQT1 patients has incomplete penetrance and show relatively mild clinical manifestations (38). MTMR4 targets early-stage endosome, regulates TGFβ signaling pathway, and thus participates in cardiovascular diseases via its FEVY domain. Smads protein family act downstream of TGFβ to play critical roles in CVDs. MTMR4 dephosphorylates Smad2/3 within early-stage endosome via binding to the phosphorylated SXS-motif of Smad2/3 and thus stabilizes TGFβ signal (40). Dysregulation of TGF-β signal pathway is involved in the development of aortic aneurysm (41). Three microRNAs (miRNAs) have been identified as diagnostic biomarkers for aortic aneurysm. MTMR4 is the same predictive target of these three miRNAs and shows negative correction with the miRNAs (42). Though MTMR4 may affect aortic aneurysm via affecting TGFβ signaling pathway, there is no direct evidence that MTMR4 has definite curable effects on aortic aneurysm. In addition, Dy et al. (2019) found that MTMR3/MTMR4 regulated interferon gene stimulating factor (STING) trafficking by regulating ptdins3p production, suggesting that MTMR3/MTMR4 may be a potential therapeutic target in the process of myocardial fibrosis, macrophage infiltration and cardiac inflammatory response in patients with diabetes and obesity mediated by STING signaling, which needs to be further experimentally verified (43).

### 3.3 MTMR7

Peroxisome proliferators-activated receptors (PPARs) belong to the nuclear receptor superfamily, among which a nuclear transcription factor PPARγ inhibits proliferation of cancer cells, exerts lipid lowering and sensitization, and is utilized to prevent against type 2 diabetes (8, 44). RAS-ERK signal transduction has multiple regulatory effects on PPARγ. Weidner et al. demonstrated that downstream effectors of RAS inhibit PPARγ, e.g., by nuclear export and cytosolic sequestration through MEK1. as well as by ERK1/2-dependent phosphorylation (45). Further research found that MTMR7 as a novel interaction partner for PPARγ to counter the inhibitory effect of RAS-ERK on PPARγ (44). MTMR7 is widely expressed in brain, muscle, liver, kidney and cytoplasmic segregation (46). Unlike other MTMRs, MTMR7 is a pro-survival phosphatase and utilizes inositol-1,3 bisphosphate (Ins(1,3)P2) as substrate (47). A synthetic peptide that mimics the CC domain of MTMR7 is able to interact with the steroid receptor coactivator (SRC1) binding site of PPARγ both *in vivo* and *in vitro*, indicating that MTMR7 interacts with PPARγ and positively regulates of PPARγ (44). Furthermore, MTMR7 is known to suppress RAS-ERK1/2 and PI3K/AKT/mTOR pathway (8), suggesting that MTMR7 can also indirectly enhance the function of PPARγ. PPARγ is involved in glucose and fatty acid oxidation in cardiac and vascular tissues (48). Pioglitazone, the agonist of PPARγ, ameliorates mitochondrial disorders, reduces lipid deposition during, and thus prevents against severe pulmonary arterial hypertension and vascular remodeling (49). Therefore, further efforts are needed to investigate whether MTMR7 can function in cardiovascular diseases via affecting PPARγ. In addition, a relatively rare SNP variant (rs4921542) in the intron region of MTMR7 is associated with a high risk of variant Creutzfeldt-Jakob disease (vCJD) (50). So, MTMR7 may be a potential research target linking vCJD and cardiovascular disease.

### 3.4 MTMR14

MTMR14, which takes a variety of phosphates as substrates, is originally identified in human centronuclear myopathy and expressed at kidney, placenta, fat, liver, teste, heart and muscle (51). Recent researches demonstrate that MTMR14 is involved in cardiovascular regulation. The cardiovascular protection mediated by MTMR14 is related to PI3K, which can be activated by G protein-coupled receptors after stress and induces AKT phosphorylation and cardiac hypertrophy (52). MTMR14 can modulate the activity of PI(3,5)P2, which is crucial for maintaining the homeostasis of in muscle (53). MTMR14-mediated regulation of PI(3,5)P2 also exerts in cardiac tissue. Chad et al. demonstrate that PI(3,5)P2 directly binds to RyR2 thus promoting the release of Ca^2+^ from sarcoplasmic reticulum and improving cardiac contractility (53).

MTMR14 is also discovered to has a specific inhibitory effect on the proliferation of vascular smooth muscle cell (VSMC). Abnormal proliferation and migration of VSMC is the critical step during the development of atherosclerosis and vascular restenosis (54). Kong et al. demonstrate that vascular injury causes neointimal formation and increased expression of MTMR14 in carotid artery (10). Knocking down MTMR14 aggravates neointimal hyperplasia by inducing proliferation of VSMC. Further analysis shows that knocking out MTMR14 (MTMR14-KO) enhances the phosphorylation of polo-like kinase 1 (PLK1), ERK and AKT. PLK1 is activated in proliferating cells and promotes proliferation by activating MEK/ERK signal (55–57). Silencing PLK1 ameliorates MTMR14-KO-induced vascular neointimal hyperplasia, indicating MTMR14 inhibits PLK1 activity by interacting with PLK1, suppresses MAPK activity and thus inhibits vascular restenosis (10).

## 4 Common biological functions of MTMRs

In addition to participating in the occurrence and development of cardiovascular events, MTMRs are also involved in some cellular processes that are critical in cardiovascular regulation. For example, MTMR9/MTMR14 involved in regulating lipid metabolism. MTMR6 negatively regulates ion channels. Additionally, some MTMRs regulate endocytosis, membrane transport during autophagy, and maintaining autophagy flow (Table 2).

### 4.1 Effect of MTMRs in regulation of metabolism

Increased prevalence and incidence of obesity have garnered considerable attention worldwide (58). Obesity typically manifests as systemic inflammation, metabolic complications, and fatty accumulation (59), leading to increased risk of chronic diseases such as cardiovascular diseases, cancer, and respiratory diseases (60). Previous studies have found that MTMR7 is highly correlated with glucose metabolism and mammalian targets of rapamycin complex 1 (mTORC1). Further experiments confirmed that MTMR7 significantly inhibited glycolysis and mTORC1 activity in PDGF BB-excited VSMCs *in vitro*, so it was concluded that MTMR7 inhibited glucose metabolism and thus inhibited VSMC proliferation and migration and vascular intimal proliferation (61). PPARγ is a member of the nuclear receptor superfamily that plays a key role in the differentiation, maintenance, and function of adipocytes (62). In addition, PPARγ also plays an important role in pulmonary hypertension, atherosclerotic and right heart failure cardiovascular disease (45, 63). MTMR7 can interact with PPAR, which indirectly indicates that MTMR7 regulates cardiovascular disease by regulating metabolism. Additionaly, Johnson et al. demonstrate that MTMR9 is located at 8p23-p22 segment and is associated with the obesity phenotype (64). Hotta et al. confirm a close relationship between body mass index (BMI) and single nucleotide polymorphism (SNP) RS2293855. Further analysis shows that the transcription level of MTMR9 in the mouse hypothalamic region is increased after fasting while decreased after high-fat diet, suggesting that genetic variants in MTMR9 may cause obesity and hypertension by regulating hypothalamic neuropeptides (65). SNP RS2293855 on the MTMR9 gene intron is associated with increased HbA1c level, insulin sensitivity, and insulin secretion. However, this association disappears after the recovery of blood glucose, indicating this association is mediated by glycaemic pathways (66). Moreover, MTMR9 overexpression can reduce the surface expression of Wnt/β-catenin signaling gene WNT3A (20). And the rs752107 polymorphism of WNT3A gene is significantly associated with susceptibility to Essential hypertension (EH), and is also associated with the risk of heart failure (HF) and ischemic stroke (IS), suggesting that MTMR9 may be a target between Wnt signaling and cardiovascular diseases (67).

In addition to MTMR9, catalytically active MTMRs also play a role in regulating lipid metabolism. The weight of adult MTMR14-KO mice increases more quickly than that of wild type mice (68), indicating that MTMR14 is involved in obesity. Further analysis shows that MTMR14 deletion results in fatty accumulation, inflammation, and metabolic disorder by releasing serum cytokines, abnormal regulation of several modulatory genes and the PI3K/AKT and ERK signaling pathways. There are also studies demonstrating that elder MTMR14-KO mice display more severe fatty accumulation and metabolic disorder, suggesting that MTMR14-mediated inflammation and metabolic disorder are age-dependent (51) (68).

### 4.2 Regulates potassium channels

There are four genes encoding Ca^2+^-activated K^+^ channels with small or intermediate conductance, including KCa2.1, KCa2.2, KCa2.3, and KCa3.1. KCa3.1 (also known as KCa4, IKCa1, hIK1, or SK4) can be activated by intracellular Ca^2+^ and the gating of KCa3.1 is voltage-independent (69). It has been reported that KCa3.1 mRNA expression was significantly increased in the coronary arteries of pigs with early atherosclerosis (70) or in rats with myocardial infarction (71) and hypertension (72). Therefore, inhibition of KCa3.1 activity is essential for the development of cardiovascular diseases. CHO-KCa3.1 is a cell line stably expressing KCa3.1. The KCa3.1 current is significantly decreased after co-transfection of CHO-KCa3.1 with GFP-labeled MTMR6, suggesting that MTMR6 inhibits the activity of KCa3.1 (73). MTM1 does not display inhibitory effect on KCa3.1, however, the chimeric MTM1 in which the CC domain is exchanged with the MTMR6 CC domain also inhibits KCa3.1, demonstrating that this inhibition is mediated by the CC domain (73). MTMR6 is known to form heterodimer with MTMR9 to exert its function (5). However, silencing MTMR6 instead of MTMR9 attenuates the lethality of *Vps34* mutation in C. elegans, indicating that MTMR6 can also function in a MTMR9-independent manner (73). Whether MTMR9 is essential for the inhibitory role of MTMR6 in KCa3.1 also needs further investigation. MTMR6 selectively dephosphorylates PI(3)P and leads to declined PI(3)P in lipid microdomains adjacent to K(Ca)3.1. Further analysis shows that KCa3.1 activity is also suppressed by PI3K inhibitors, and this suppression can be reversed by the supplement of PI(3)P instead of other phosphoinositides. Additionally, MTMR6-mediated inhibition of K(Ca)3.1 is also rescued by the supplement of PI(3)P (73). Taken together, these data suggest that MTMR6-mediated inhibition of K(Ca)3.1 by dephosphorylating and decreasing PI(3)P may participant in the development of some cardiovascular diseases.

### 4.3 Role of MTMRs in autophagy/apoptosis

Autophagy and apoptosis are closely regulated processes in cellular and tissue homeostasis, development, and disease (74). Autophagy is a evolutionarily conserved cellular process that depends on lysosomal degradation of cytoplasmic components (75). Serving as an important cellular survival mechanism under stress, autophagy plays a critical role in maintaining cellular homeostasis and function (76). Autophagy or necessary proteins involved in the autophagy process may promote cell death, either by decomposing cells to promote apoptosis or by activating the necrosis program to promote cell death (77). So autophagy is inseparable from programmed cell death. There are many lines of evidences that autophagy/apoptosis is involved in the regulation of CVDs, such as atherosclerosis, hypertension, myocardial infarction, and cardiomyopathy, etc. (78, 79). Depending on PI(3)P- and PI(3,5)P2-mediated degradation of membranes during autophagosome-lysosome fusion, macromolecules are digested by lysosomal enzymes and transported to the cytoplasm for anabolic activities (80). Therefore, enzymes that facilitate the conversion or production of PIs are important for autophagy. MTMRs are known to modulate membrane trafficking (81) and maintain autophagic flux (82) during autophagy and endocytosis. For instance, suppression of MTM1, MTMR1, MTMR2, and MTMR3 in mammal cells, zebrafish, mice, and fruit flies both increase the amount of autophagosome (83–85). And Overexpression of MTM1 can inhibit granulosa cell proliferation and promote cell apoptosis in polycystic ovary syndrome (86). MTMR3 is one of the main genes involved in the regulation of autophagy pathway in mammalian cells, and MTMR3 induces autophagy by inducing or down-regulating mTORC1 (87), while up-regulation leads to the reduction of autophagosomes, thereby inhibiting autophagy (36, 88, 89). MTMR2/MTMR5 is a heterodimer that suppresses autophagy and is crucial for autophagy initiation and autophagosome maturation (90). MTM1, MTMR6 and MTMR9 in C. elegans promote fluid-phase endocytosis. Moreover, Allen et al. demonstrate that CG3530 (dMTMR6), which affects autophagy in fruit flies, is homologous to the human MTMR6, exerts as a regulator of autophagy flux in Drosophila cells, and shares similar function to MTMR8 in mammal cells (82). Further analysis shows that dMTMR6 and MTMR8 function as positive regulators of autophagosome-lysosome homeostasis and positively regulate late autophagy. Downregulation of dMTMR6 and MTMR8 leads to accumulation of autophagic vesicles and disorder of phagocytosis, which finally impairs lysosomal homeostasis (82). In spite of the effect on promoting late autophagy, MTMR6 also exerts as an antagonism under stress via interference with PI3K signal pathway and inhibiting formation of autophagosome (4). There have also been reports that deletion of either the MTMR6 or the MTMR8/MTMR9 complex leads to an increase in autophagy (43, 91). Wang et al. found that negative regulation of MTMR6 can inhibit the proliferation of ovarian cancer cells and promote apoptosis. MTMR7 and MTMR8 are homologous to MTMR6 and can also interact with MTMR9 (19, 46). MTMR7 inhibits insulin signaling and negatively regulates autophagy in colorectal cell line (8), while MTMR8/MTMR9 regulates the PI(3)P pool and positively modulates the level of p62, whose degradation within autophagosome serves as a hallmark of autophagy. However, silencing MTMR8 or MTMR9 alone does not affect autophagy (19). MTMR14 is able to suppress basal autophagy instead of stress-induced autophagy (4). Knocking down MTMR14 leads to accumulation of autophagosome and increased level of LC3Ⅱ, which prevents the subsequent degradation of autophagy macromolecules and provides evidence that MTMR14 is a positive regulator of autophagy (92). MTMR14 can also regulate cardiomyocyte enlargement and programmed cell death through the PI3K \/AKT pathway, as well as inhibit nuclear transcription factor (NF)- *κ* B) signal transduction reduces cell death and inflammatory response, serving as a protective factor against hepatic ischemia-reperfusion injury. Moreover, knocking out MTMR14 can promote tumor cell apoptosis and inhibit cell migration.

## 5 Conclusion

**Figure 2:**
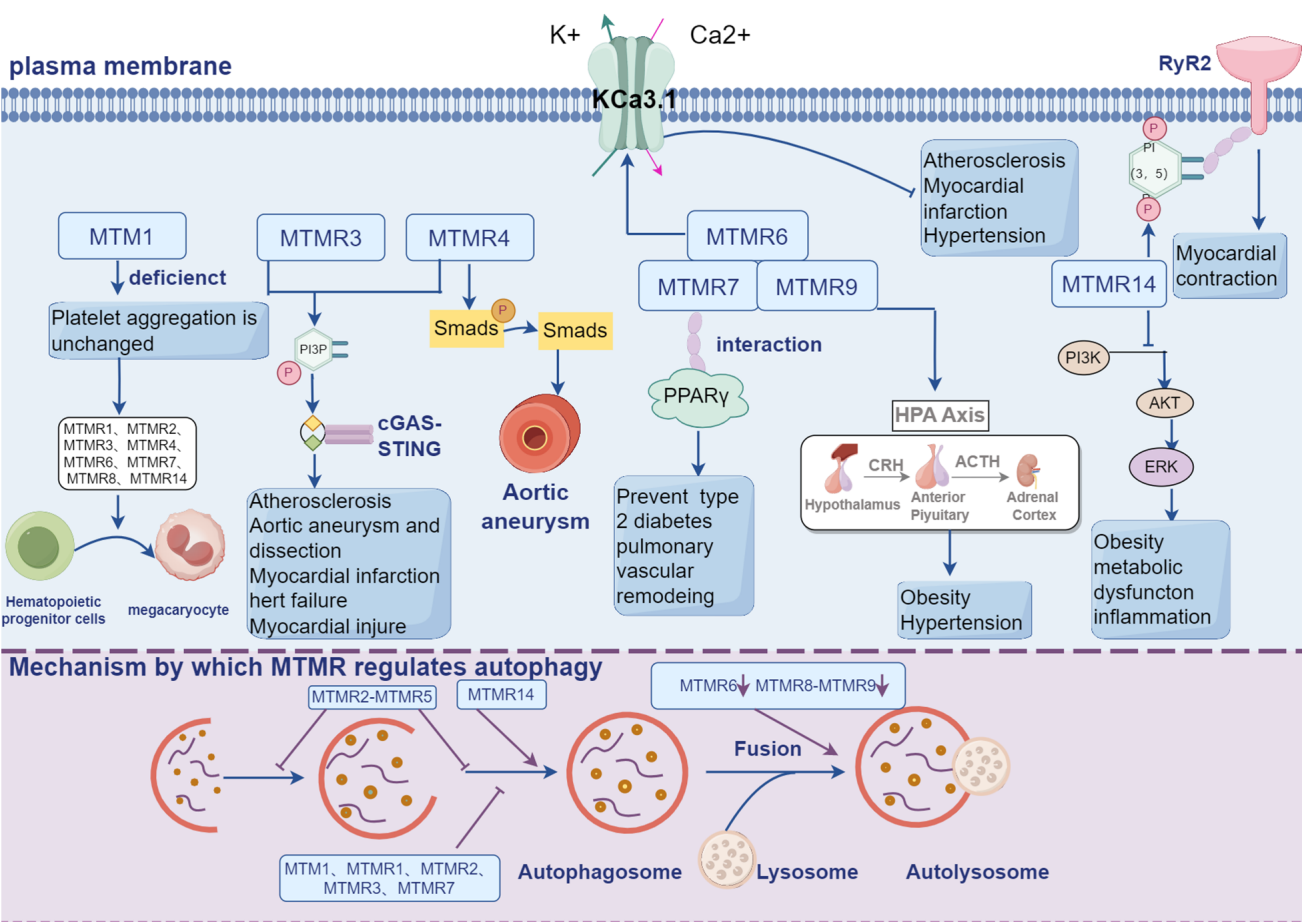
MTMRs not only exert their regulatory effects in the cardiovascular system through the PI3K-AKT signaling pathway, but also participate in a series of cellular processes crucial for cardiovascular regulation, including autophagy, metabolism, and ion channels.

The current review summarized the roles and mechanisms of MTMRs in CVDs. MTMRs are a kind of phosphatases that are involved in many biological processes. Mutations in MTMRs are associated with several lines of diseases, including neural disorders, skeletal muscle defects, cancers and CVDs. The canonical biological roles of MTMRs include forming homodimer or heterodimer and regulating PI3K/AKT pathway, by which MTMRs exert their regulatory roles in cardiovascular system. In addition to directly participating in the development of cardiovascular events, MTMRs are also involved in a series of cellular processes that are critical in cardiovascular regulation, including autophagy, metabolism and ion channel (Figure 2). However, the current opinions on MTMRs-dependent cardiovascular regulation are still insufficient. For example, since several MTMRs regulate proliferation in cancer cells and neurocytes, what about the role of these MTMRs in the proliferation of VSMCs, vascular adventitial fibroblasts and vascular intimal hyperplasia? Do MTMRs modulate the development of CVDs via regulating mechanism, autophagy or potassium channels? Taken collectively, a better understanding of the functions of MTMs/MTMRs might significantly contribute to develop novel targets for preventing CVDs.
